# A metastasis-on-a-chip approach to explore the sympathetic modulation of breast cancer bone metastasis

**DOI:** 10.1016/j.mtbio.2022.100219

**Published:** 2022-02-14

**Authors:** Francisco Conceição, Daniela M. Sousa, Joshua Loessberg-Zahl, Anke R. Vollertsen, Estrela Neto, Kent Søe, Joana Paredes, Anne Leferink, Meriem Lamghari

**Affiliations:** a Instituto de Investigação e Inovação Em Saúde (I3S), Universidade Do Porto, 4200-135, Porto, Portugal; b INEB—Instituto Nacional de Engenharia Biomédica, Universidade Do Porto, 4200-135, Porto, Portugal; c ICBAS—Instituto de Ciências Biomédicas Abel Salazar, Universidade Do Porto, 4050-313, Porto, Portugal; d IPATIMUP—Instituto de Patologia e Imunologia Molecular da Universidade Do Porto, 4200-135, Porto, Portugal; e FMUP—Faculdade de Medicina da Universidade Do Porto, 4200-319, Porto, Portugal; f BIOS Lab on a Chip Group, MESA+ Institute for Nanotechnology, Max Planck - University of Twente Center for Complex Fluid Dynamics, University of Twente, P.O. Box 217, 7500, AE Enschede, the Netherlands; g Applied Stem Cell Technologies, TechMed Centre, University of Twente, PO Box 217, 7500, AE, Enschede, the Netherlands; h Clinical Cell Biology, Vejle Hospital/Lillebaelt Hospital, Department of Regional Health Research, University of Southern Denmark, 7100, Vejle, Denmark

**Keywords:** Metastasis-on-a-chip, Breast cancer, Bone metastasis, Sympathetic nervous system, Paracrine, PDMS, poly-dimethylsiloxane, SNS, Sympathetic Nervous System, NE, norepinephrine, TH, tyrosine hydroxylase, MCP-1, monocyte chemoattractant protein 1, IL-6, interleukin 6, MIP-1α, macrophage inflammatory protein 1α, IL, interleukin

## Abstract

Organ-on-a-chip models have emerged as a powerful tool to model cancer metastasis and to decipher specific crosstalk between cancer cells and relevant regulators of this particular niche. Recently, the sympathetic nervous system (SNS) was proposed as an important modulator of breast cancer bone metastasis. However, epidemiological studies concerning the benefits of the SNS targeting drugs on breast cancer survival and recurrence remain controversial. Thus, the role of SNS signaling over bone metastatic cancer cellular processes still requires further clarification. Herein, we present a novel humanized organ-on-a-chip model recapitulating neuro-breast cancer crosstalk in a bone metastatic context. We developed and validated an innovative three-dimensional printing based multi-compartment microfluidic platform, allowing both selective and dynamic multicellular paracrine signaling between sympathetic neurons, bone tropic breast cancer cells and osteoclasts. The selective multicellular crosstalk in combination with biochemical, microscopic and proteomic profiling show that synergistic paracrine signaling from sympathetic neurons and osteoclasts increase breast cancer aggressiveness demonstrated by augmented levels of pro-inflammatory cytokines (e.g. interleukin-6 and macrophage inflammatory protein 1α). Overall, this work introduced a novel and versatile platform that could potentially be used to unravel new mechanisms involved in intracellular communication at the bone metastatic niche.

## Graphical abstract

The sympathetic nervous system is an important modulator of breast cancer bone metastasis. Here, a novel three-dimensional printing based, multi-compartment metastasis-on-a-chip platform with controllable diffusion directionality is described, to model the interactions on breast cancer bone metastatic niche. The multicellular crosstalk between human sympathetic neurons, breast cancer cells, and osteoclasts increases cancer cell expression of pro-inflammatory cytokines (IL-6 and MIP-1α).

**Figure:**
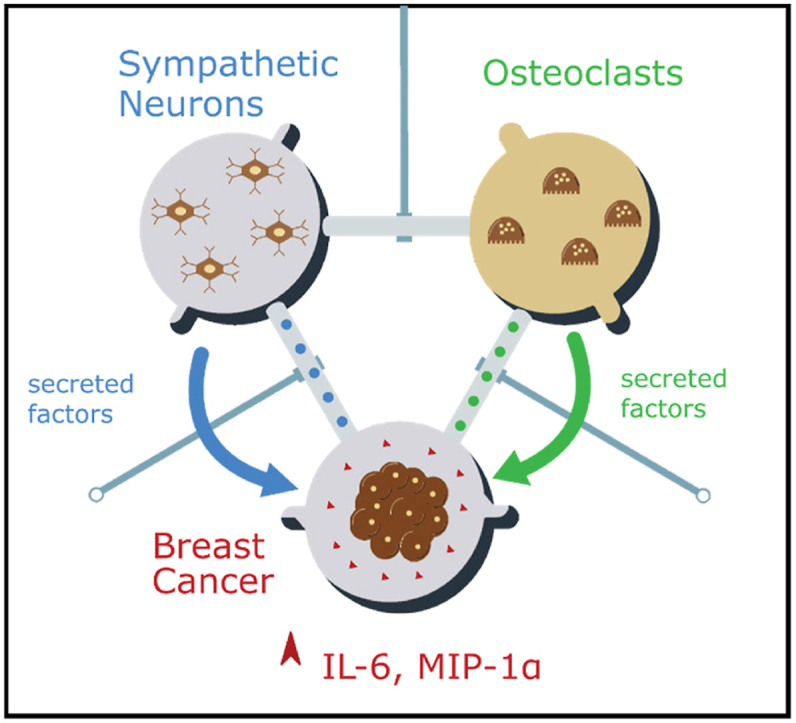

## 1 Introduction

Breast cancer bone metastasis is an complex process that encompasses cell extravasation from the circulatory system into the bone, engraftment on a suitable niche, escape from dormancy, proliferation and uncoupling of the bone remodeling to fuel tumor growth [1]. Bone is the most common site of metastasis in breast cancer. Within the bone, breast cancer cells over-activate bone resorbing osteoclasts and shift the physiological balance in bone remodeling towards increased bone destruction. This leads to severe skeletal complications, such as bone pain, hypercalcemia and bone fractures [1]. The elucidation of the cellular and molecular mechanisms by which breast cancer cells engraft and proliferate in the bone niche is, therefore, of crucial importance to improve the available therapeutic options. However, several barriers still hamper the study of the metastatic bone niche. In vivo models, which are able to recapitulate the complexity of the human disease, are limited and of difficult execution, whereas the dissection of specific signaling pathways involved in bone metastasis progression is extremely complex. Furthermore, high mortality rates and pain associated with the in vivo modelling of this specific disease inherently raises ethical constraints that limit the use of animal models. On the other hand, classical in vitro models are simplistic and do not replicate the native features of the bone microenvironment.

Microfluidic tools have emerged in the past decade as an alternative to conventional in vitro and in vivo models, since these combine three dimensional (3D) matrices with human cells while allowing a fine control over spatial and temporal parameters of culture [2]. In addition, fluidic connection of different cell compartments as well as the control of flow and shear facilitates more physiologically relevant modelling [3]. Microfluidic platforms have in the past been already used as models for bone cancer processes including: i) selective tropism of myeloma and breast cancer cells towards bone cells [2,4]; ii) extravasation of breast cancer cells from circulation into extracellular matrix (ECM) structures based on collagen or fibrin [[5], [6], [7], [8]]; iii) colorectal, myeloma and breast cancer cell engraftment and proliferation in mineralized matrices [[9], [10], [11], [12]]; iv) cancer drug screening and toxicity assessment [3,13]. Thus, microfluidic technology can tackle constraints associated to standard in vitro tools in the study of the crosstalk occurring during breast cancer bone metastasis, and thus improve our knowledge on the signaling pathways governing the metastatic process.

Despite the numerous advantages of metastasis-on-a-chip in vitro tools, the typical photolithography processes used in their fabrication require expensive infrastructure and highly skilled personnel. 3D printing is becoming a viable alternative for microfluidic fabrication since it combines accessibility of standard benchtop 3D printers and a high degree of design freedom which is not trivial to achieve via photolithography [14]. Furthermore, advances in printer technology have improved surface roughness of 3D printed template molds, to the point that the resulting prototypes become compatible with plasma sealing procedures [15]. 3D printing is also suited for valve fabrication, which allows the control of flow resistance and diffusion through the different fluidic compartments [16].

The sympathetic nervous system (SNS) was brought to light as a potential therapeutic target for the treatment of breast cancer due to several findings in pre-clinical and epidemiologic studies [[17], [18], [19]], which correlated sympathetic hyperactivity and poor patient prognosis. However, the beneficial effect of SNS targeting drugs on the treatment of breast cancer remains controversial, since other reports failed to replicate such correlations [20,21]. It is well established that the SNS acts on multiple cellular targets throughout the body, mainly via the release of norepinephrine (NE) by sympathetic nerve endings and through systemic release of epinephrine into circulation. Functional studies demonstrated that the sympathetic stimulus is able to increase breast cancer circulating tumor cell retention and extravasation to the bone [22]. Nonetheless, dissection of sympathetic signaling in the context of human breast cancer bone metastasis was not yet reported and the mechanisms governing breast cancer cell response to sympathetic input within the bone microenvironment are still poorly understood.

As stated above, microfluidic tools offer multiple advantages regarding standard in vitro models such as compartmentalization and fine tuning of culture parameters. We have previously established models of neuronal/non-neuronal cellular communication in compartmentalized microfluidic devices to address sensory innervation in the bone microenvironment [23,24]. However, to date there are no microfluidic models described for the study of sympathetic stimuli on the breast cancer bone metastatic niche.

In this study, we have designed and prototyped a new 3D printing based metastasis-on-a-chip platform to reproduce the effect of sympathetic activation on the dynamic crosstalk that occurs between breast cancer cells and bone cells in a fully humanized model. Our platform combines three different human cell types: 1) a bone tropic breast cancer cell variant, 2) sympathetic neurons and 3) human peripheral blood derived osteoclasts seeded on top of a bone matrix. The microfluidic platform was specially designed to physically separate the cells into different compartments to facilitate the identification of secreted factors involved in intercellular communication while preventing direct cell-cell interactions. Furthermore, inclusion of fluidic flow between different compartments allow a unidirectional communication from one compartment to the remaining ones. Our metastasis-on-a-chip platform is based on static diffusion in order to facilitate bidirectional communication between each compartment. Additionally, our platform also allows the manipulation of communication between the different compartments through the use of incorporated pressure actuating valves. We were able to successfully optimize the culture of each cell type and demonstrated that the dynamic interaction between neurons, breast cancer cells and osteoclasts translates into an increased pro-inflammatory phenotype. In addition, manipulation of the communication between compartments allowed us to show that direct neuronal stimulation of osteoclasts is not required to observe inflammatory cytokine upregulation. Based on these results, we believe that our versatile platform can be a potential tool for fundamental research on multiple research topics. The use of widely accessible 3D printing technology further highlights the adaptability of our metastasis-on-a-chip platform.

## 2 Materials and methods

### 2.1 Fabrication and assembly of the metastasis-on-a-chip platform

Each microfluidic component was made out of poly-dimethylsiloxane (PDMS, Sylgard 184, Dow Corning) using specially designed 3D printed molds. Molds were designed using SolidWorks (Dassault Systèmes) and 3D printed in a Form 3 printer (Formlabs) with a Grey V4 resin (Formlabs). These molds were post-processed by two rounds of immersion in isopropanol for 15 ​min to remove uncured resin, followed by air-drying and a heat treatment of 3 ​h at 60 ​°C. PDMS was then cast into the mold with a 10:1 (w/w) ratio of base and curing agent and thermally cured for 1 ​h 30 ​min at 60 ​°C. Each PDMS slab was separated from the mold and cleaned with residue-free tape until plasma treatment.

The microfluidic platform was designed for single use and is composed of three different structural parts bonded together: a top slab containing patterned cell compartments and diffusion channels, a bottom slab containing valve structures and a simple membrane in between. Top slabs have three equidistant compartments 6 ​mm in diameter for cell culturing (each with two medium inlets) which are interconnected through 4.5 ​mm long semi-circular channels 300 ​μm wide and 150 ​μm high. The bone and cancer compartment are 1.2 ​mm deep to accommodate the bone slice (400 ​μm thick) and the spheroid, while the Neuronal compartment is 600 ​μm deep.

PDMS membranes were produced by spin coating 1.6 ​mL PDMS at a 10:1 (w/w) ratio of base and curing agent on top of a perfluorodecyltrichlorosilane coated silicon wafer (10 ​cm diameter) with an initial spinning step at 500 ​rpm for 15 ​s and 100 ​rpm/s acceleration, followed by a second step at 1500 ​rpm for 75 ​s and 1000 ​rpm/s acceleration. The membranes were then thermally cured for 1 ​h 30 ​min at 60 ​°C.

Metastasis-on-a-chip platform assembly was achieved by covalent bonding of the different components. The membrane and cell compartment slab were first covalently bound together through oxygen plasma treatment for 1min on a Zepto Plasma Cleaner (Diener Electronic). The membrane was then cut along the contour of the PDMS slab using a scalpel and lifted from the silicon wafer. Medium inlets and pressure inlets were then punched out of the bonded membrane and the cell compartment slab using a 1 ​mm biopsy puncher (Kai Medical). The resulting structures were then bonded to the valve structure slab by oxygen plasma treatment as described previously. Right after treatment and before bonding, bovine bone slices (boneslices.com, Denmark) were placed in the bone cell compartment. Both slabs were then bonded together. Each microfluidic unit was sterilized with 70% ethanol, washed thrice with phosphate buffered saline (PBS) and equilibrated in complete medium. Neuronal compartments were incubated in a solution of 5 ​μg/mL laminin (Sigma-Aldrich) in DMEM/F12 medium (Gibco) with 10% FBS and 1% penicillin/streptomycin (Pen/Strep, Gibco) (DMEM/F12 complete medium) for 2 ​h at 37 ​°C. Compartments were washed twice with DMEM/F12 complete medium and kept at 37 ​°C until cell seeding.

### 2.2 Osteoclast isolation

Human CD14^+^ monocytes were isolated from buffy coats of healthy female blood donors as previously described [25]. Briefly, Peripheral Blood Mononuclear Cells (PBMCs) were separated using gradient centrifugation in Ficoll-Paque Plus (GE Healthcare). PBMCs were then ressuspended in 0.5% Biotin-free Bovine Serum Albumin (BSA, Sigma-Aldrich) and 2 ​mM EDTA in PBS, incubated in BD IMagTM anti-human CD14 magnetic particles (BD-Biosciences) and magnetically separated according to manufacturer's instructions. CD14^+^ cells were seeded in T75 flasks in α-MEM (Gibco) supplemented with 10% FBS, 1% Pen/Strep and 25 ​ng/mL of recombinant human macrophage colony stimulating factor (rhM-CSF, R&D Systems) at 5% CO_2_ at 37 ​°C in a humidified incubator for 2 days. Cells were then differentiated into mature osteoclasts by supplementing the medium with 25 ​ng/mL *M*-CSF and receptor activator of NF-κB ligand (RANKL, R&D Systems) for further 7 days of culture, changing medium twice.

### 2.3 Breast cancer cell spheroids

MDA-MB-231-BoM 1833 human breast carcinoma cell line (MDA-1833 henceforth), a bone tropic variant of the MDA-MB-231 ​cell line, was obtained from Dr. J. Massagué (Memorial Sloan-Kettering Cancer Center, New York). MDA-1833 ​cells were expanded in DMEM High Glucose (Gibco) with 10% FBS and 1% Pen/Strep (DMEM complete medium) at 37 ​°C and 5% CO_2_ in a humidified incubator, changing medium twice a week until reaching 80% confluence. Cells were then trypsinized (0.25% w/v trypsin, 0.1% w/v glucose and 0.05% EDTA in PBS, Life Technologies), seeded at a density of 10 ​000 ​cells per well on round bottom ultra-low adhesion 96-well plates (Corning) and incubated for 4 days in DMEM complete medium with 2.5% Matrigel Basement Membrane Matrix (Corning) to induce formation of cell spheroids.

### 2.4 Neuronal-like cell differentiation

SH-SY5Y (ATCC) cells were used as a model of human sympathetic neurons since these cells were previously reported to be able to produce NE [26]. SH-SY5Y cells were expanded in DMEM/F12 Complete medium at 37 ​°C and 5% CO_2_ in a humidified incubator, changing medium twice a week until reaching 80% confluence. Cells were then trypsinized and 20 ​000 ​cells were seeded in the laminin coated neuronal compartments and incubated at 37 ​°C overnight. Differentiation was induced by Opti-MEM medium (Gibco) supplemented with 0.5% FBS, 1% Pen/Strep and 0.1 ​μM Retinoic Acid over the course of one week, changing medium every day.

### 2.5 Metastasis-on-a-chip cell seeding

Microfluidic experiments were set up during the course of 10 days. In order to isolate compartments before cell seeding and ensure full physical separation of the different cell types, compartments were sealed off by closing the valves with a pressure of 600 ​mbar using a FlowEZ 2000 ​mbar pressure controller module (Fluigent). SH-SY5Y cells were seeded on the neuronal compartment as previously described and differentiated for 7 days with the valves open. At day 7, medium from the bone compartment was changed for α-MEM supplemented with 0.5% FBS, 1% Pen/Strep and 25 ​ng/mL rhM-CSF (Osteoclast Medium) and the medium from the cancer compartment was changed for DMEM High Glucose supplemented with 0.5% FBS and 1% Pen/Strep. Differentiated osteoclasts were then detached with Accutase (Gibco) for 10 ​min at 37 ​°C, centrifuged and seeded on the bone slice at a density of 75 ​000 ​cells in Osteoclast Medium, being left to adhere for 4 ​h. MDA-1833 individual cell spheroids were transferred to the cancer compartment, one spheroid per microfluidic device, and the platform was incubated at 37 ​°C for 3 days to allow bone resorption to occur. When required, valves were closed with a pressure of 600 ​mbar throughout the experiment. The bone compartment was supplemented with fresh 50 ​ng/mL rhM-CSF and rh-RANKL daily. After 3 days, experiments were ended and conditioned medium was collected from each compartment while keeping the valves closed. In addition, one replicate from each condition was used for immunocytochemistry: medium was removed and the compartments were washed with PBS twice before immunocytochemistry.

### 2.6 Immunocytochemistry

Cells in the microfluidic platform were fixed in 4% paraformaldehyde for 10 ​min at RT followed by 3 steps of washing with PBS and blockage of unspecific staining in a blocking solution of 5% FBS, 5% Horse Serum (Invitrogen) and 0,25% Triton X-100 (Sigma-Aldrich) in PBS for 1 ​h at 37 ​°C. Samples were then incubated with primary antibodies overnight at 4 ​°C (mice *anti*-βIII Tubulin 1:2000 [Promega]; rabbit anti-TH 1:100 [Merck]; mouse *anti*-CATK 1:100 [Santa Cruz Biotechnology]; rabbit anti-CD49f [Sigma-Aldrich]). After incubation, samples were washed thrice with PBS and labelled with secondary antibodies accordingly (Invitrogen, 1:1000 dilution) together Flash Phalloidin™ (Biolegend) for actin staining, for 1 ​h at RT. Finally, cells were washed thrice with PBS and counterstained with DAPI (1:1000 dilution), washed again to remove excess DAPI and kept at 4 ​°C until imaging. Images were acquired in a Leica SP5 confocal microscope at a resolution of 1024 ​× ​1024 pixels and z-step of 5 ​μm. Brightness was adjusted for better visualization and z-projections as well as artificial cell coloring were performed using ImageJ.

### 2.7 Flow cytometry

MDA-1833 ​cell spheroids were removed from the metastasis-on-a-chip platform and dissociated with Accutase at 37 ​°C for 20 ​min in a microtube, pipetting up and down every 5 ​min. At least 10 spheroids were pooled for each condition in one independent experiment. A commercial kit for Annexin V-APC staining (BD Pharmigen) was used according to manufacturer's instructions, but only using 1 ​μL of Annexin V and 3 ​μL Propidium Iodide. Immunostaining was performed in 1X Binding Buffer and quantified using a FACS CANTO II (BD Immunocytometry Systems) and FlowJo™ software (BD).

### 2.8 Enzyme-linked immunosorbent assays (ELISA)

Conditioned medium was collected from each compartment by closing the respective valves and aspirating the medium. Conditioned medium was centrifuged at 4 ​°C at 400 ​g for 5 ​min to remove cellular debris, transferred to a new microtube and frozen at −80 ​°C until quantification. NE was quantified by ELISA (Abnova) from conditioned medium collected and pooled from SH-SY5Y monoculture controls from three independent experiments. Quantification was performed according to manufacturer's indications, but 300 ​μL of conditioned medium was used in each replicate and a 1 ​ng/mL standard was added so that our samples would fit in the calibration curve. Protein levels of each sample were quantified using the DC Protein Assay (Bio-Rad) according to the manufacturer's instructions and were used to normalize differences between conditions.

The levels of interleukin 11 (IL-11) was quantified by ELISA (R&D Systems) from breast cancer and bone compartments of the metastasis-on-a-chip platform according to manufacturer's indications. Medium was diluted 10x before quantification to fit the calibration curve and samples were normalized using the total protein levels as mentioned above.

The levels of interleukin 6 (IL-6) and macrophage inflammatory protein 1α (MIP-1α) were similarly quantified by ELISA (Sigma-Aldrich) from the breast cancer compartments of the microfluidics (unless otherwise stated) according to manufacturer's instructions. Medium was diluted 90x before quantification to fit the calibration curve and samples were normalized using the total protein levels.

### 2.9 Quantification of bone resorption

Osteoclast resorption events were stained with toluidine blue (Sigma-Aldrich). The entire bone surface was analysed using a G50 100 graticule (Pyser Optics) installed on the ocular of an BH-2 optical microscope (Olympus) as previously described [27]. The total number of events present throughout the bone surface were counted using the graticule as a frame (using a total of 16–17 graticules per bone slice). Individual resorption events were divided in two resorption types, pits and trenches. Pits are single, circular excavations with well-defined edges while trenches are elongated and continuous grooves with a length/width ratio equal or greater than two [28]. The percentage of trenches per total events was used to compare individual experiments independently of eroded surface variations. Samples were blinded before eroded surface quantification by one researcher.

### 2.10 Proteomic analysis

Conditioned medium from four MDA-1833 spheroids cultured either on the metastasis-on-a-chip platform or in 96 well-plates was pooled and centrifuged at 300 ​g for 5 ​min to pellet cellular debris. Conditioned media was then transferred to micro tubes and protein concentration was measured as described above. 50 ​μg of protein from each condition was processed using the solid-phase-enhanced-sample-preparation (SP3) protocol, as previously described [29], followed by enzymatic digestion overnight with trypsin/LysC (2 ​μg) at 37 ​°C and 1000 ​rpm.

Protein identification was carried out by nano Liquid Chromatography coupled with Mass Spectrometry (LC-MS/MS) and data was analysed with Proteome Discoverer software (Thermo Scientific) as described by Osório et al. [30]. The ratio between the protein abundances in the conditioned medium from microfluidic and well plates was used to compare between both conditions.

### 2.11 Protein array

After tri-culture in the microfluidic platform, cancer compartment conditioned medium from three independent experiments was pooled and screened for proteins involved in bone metabolism using the G-Series Human Bone Metabolism Array 1000 (RayBiotech) according to manufacturer's instructions. Briefly, the arrays were blocked for 30 ​min and incubated with 100 ​μL of sample overnight at 4 ​°C, followed by incubation with biotinylated antibody cocktail for 4 ​h at room temperature. The slides were then incubated with Cy3 conjugated streptavidin for 1 ​h in the dark at room temperature. After washing steps, droplets were removed using a compressed argon stream. Slides were sent to the supplier to be imaged (Tebu-Bio). Array data was analysed using the Spotxel® software (Version 2.2.2, SICASYS Software GmbH) and the GAL file supplied by the manufacturer. After alignment with the GAL file, data was extracted using the original image without changes in intensity values. Quantification of the intensity values was performed by Flex-Spot Detection method and with noise filtering and local background correction method. Extracted intensity values were then analysed using the Excel analysis tool supplied by the manufacturer. After intra-assay normalization, intensity values were normalized for protein content, values for the culture medium alone were subtracted to each sample and fold-changes relative to controls were calculated.

### 2.12 Statistics

All experiments were performed at least three times. One-way ANOVA test followed by Holm-Sidák's multiple comparison test was used to assess statistical significance between conditions. When two conditions were being compared, nonparametric Mann-Whitney tests were used. Differences between groups were considered significant when ∗p ​< ​0.05, ∗∗p ​< ​0.01, ∗∗∗p ​< ​0.001. Data analysis was performed using GraphPad Prism software v.9.1.0 for Windows (GraphPad Software).

## 3 Results

### 3.1 Bone metastasis-on-a-chip design

The aim of this study was to establish a model that would allow us to clarify how breast cancer cells respond to sympathetic stimuli in a bone metastatic context, specifically focusing on the contribution of cell-secreted factors. In order to achieve this, the versatility of microfluidic platforms was appealing, since these would allow study of complex interactions between cancer cells and other significant cell components in the metastatic process, in a fully humanized system. Instead of using standard photolithography for the production of microfluidic devices, we took advantage of 3D printing to be able to quickly prototype our molds in a cost-effective fashion, while maintaining an adequate resolution (smallest feature of the mold is 150 ​μm while the minimum laser spot size of the 3D printer is 85 ​μm).

**Fig. 1:**
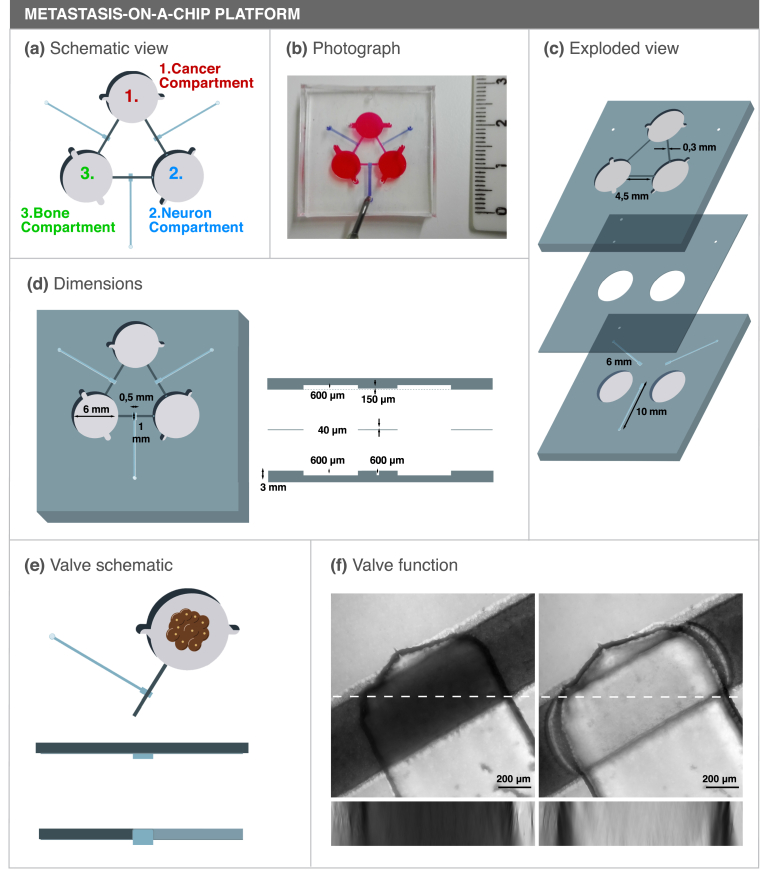
Concept and design of a novel microfluidic platform with three interconnected culture chambers. (a) Schematic representation of the microfluidic platform and (b) photograph of an assembled microfluidic colored with food dyes. (c) Exploded view of the three components of the microfluidic platform. (d) Top (left) and side (right) view of the microfluidic platform. (e) Schematic view of a functioning valve. When pressure is applied in the valve channel, the flexible PDMS membrane occludes the diffusion channel, blocking the communication between compartments. (f) Valve segment z-projection of an open valve (left) or closed valve (right) after applying a 600 ​mbar pressure on the valve channel. The microfluidic compartments were filled with toluidine blue dye and a 250 ​μm stack was obtained on a confocal microscope. An XZ orthogonal view is also shown below each respective image (corresponding to the dashed line). Scale bar 200 ​μm.

Our microfluidic chip was designed to compartmentalize three different cell types with no direct cell-cell contact but still allow diffusion dependent chemical communication between compartments (Fig. 1a and b). Importantly, we are able to dictate the direction of communication between compartments by using Quake valves incorporated in the metastasis-on-a-chip design. This microfluidic chip is composed of three different structural parts bonded together: a top slab containing patterned cell compartments and diffusion channels, a bottom slab containing valve structures and a simple membrane in between (Fig. 1c).

The ability to close the diffusion channels is an important feature both for cell seeding and to explore indirect routes of communication between different compartments. With that in mind, valve structures 500 ​μm wide and 1 ​mm long were included in the center of the diffusion channels to block the communication between compartments when desired (Fig. 1d). Three Quake valves were included on our microfluidic platform, one on each diffusion channel. Pressure applied on each valve channel will push the flexible 40 ​μm thick PDMS membrane located between the main PDMS slabs, closing the diffusion channels (Fig. 1e and f). To test the valves, the diffusion channels were filled with Toluidine Blue dye and a pressure of 600 ​mbar was applied to the valve channel. Micrographs of the valve section show that there was no dye in the diffusion channel when the valves were in a closed state (Fig. 1f, right) while dye was observed in the diffusion channel when the valves where in an open state (Fig. 1f, left). Similarly, 5 ​kDa fluorescent-labelled Dextran was not able to diffuse through a closed valve, further validating their functionality (Fig. S1). Furthermore, no diffusion was observed from one compartment to the other when all the valves were closed after three days of incubation, which was a relevant timeframe for our cell culture setup (Fig. S1). The closure of the valves was reversible (Supplementary movie 1) and the valves maintained their function over 20 cycles of opening and closure without rupturing (data not shown).

As already mentioned, current in vitro models fail to replicate the three-dimensional features of in vivo bone, which has profound biological and biomechanical implications in osteoclast biology. To circumvent that limitation, mineralized bone ECM preserving the structural and biological cues of in vivo bone were included in our model. Furthermore, to hamper the migration of osteoclasts and breast cancer cells from each respective compartment, a spatial offset between the bone and cancer compartment floor and the diffusion channels was incorporated in the design (Fig. 1d, right panel). This was not the case for the neuronal compartment to allow neuronal cells to elongate their axonal extensions into the diffusion channels and maximize the dissemination of neuronal factors to the other compartments.

### 3.2 Cancer compartment: 3D culture and proteomic analysis

To form bone metastasis, disseminated breast cancer cells acquire a specific set of characteristics that are distinct from the primary tumor [31]. Accordingly, bone tropic breast cancer cell (MDA-1833) spheroids were used as a model of breast cancer cells that are more prone to establish metastasis in the bone, which have been previously characterized [31] and are shown to be biologically relevant for the study of bone metastasis. In addition, breast cancer spheroids are commonly used to replicate the 3D features of in vivo tumors [32].

**Fig. 2:**
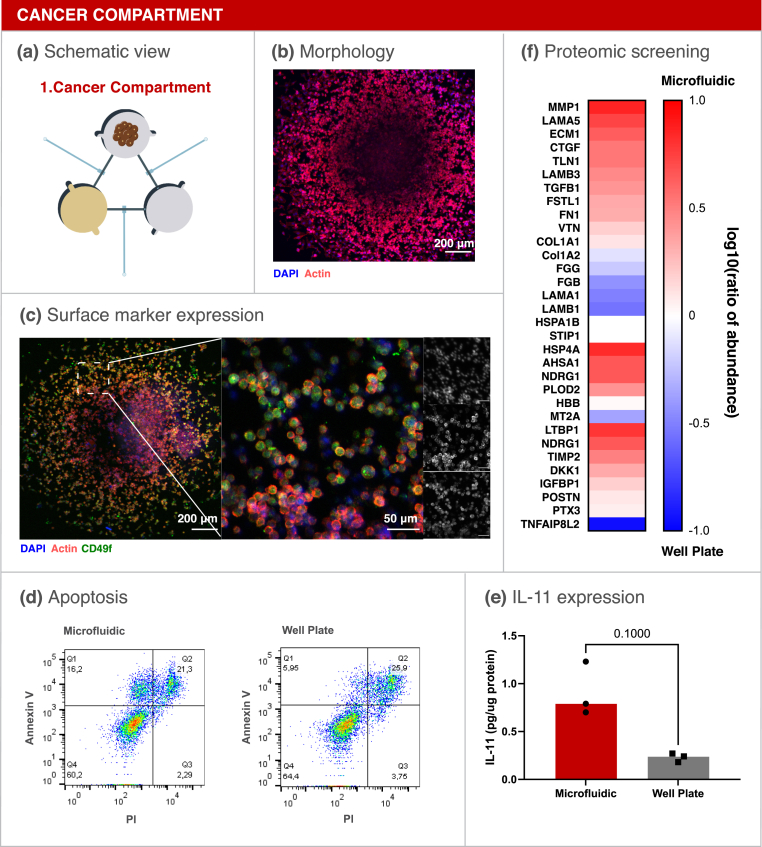
Breast Cancer compartment optimization. (a) Schematic representation of the breast cancer compartment. (b) Micrograph of a MDA-1833 ​cell spheroid cultured for 3 days on the microfluidic platform. Blue - DAPI. Red - F-actin. Scale bar - 200 ​μm. (c) Expression of CD49f on MDA-1833 ​cells. DAPI (blue), F-Actin (red) and CD49f (green). Scale bar - 200 ​μm. Inset single channel images are shown on the right DAPI (blue, top), F-Actin (red, mid) and CD49f (green, bottom). Inset scale bar - 50 ​μm. (d) Annexin V quantification by flow cytometry of breast cancer spheroids cultured inside the microfluidic platform (left) or in standard well plates (right). Ten spheroids were pooled together for the analysis. (e) IL-11 quantification in conditioned media from MDA-1833 spheroids cultured in the microfluidic platform or in well plates. Data is expressed as median of individual data points from 3 independent experiments and was normalized to the total protein content (Mann-Whitney test, p ​= ​0.1000). (f) Proteomic screening of the conditioned media from MDA-1833 spheroids cultured in the microfluidic or in standard well plates. Data is represented as the logarithm of base 10 of the ratio between the abundance of each secreted protein within the microfluidic and well plate.

Spheroids were introduced in the cancer compartment and cultured for 3 days inside the platform (Fig. 2a). First, cell morphology was assessed by F-actin staining of MDA-1833 spheroids inside the microfluidic compartment (Fig. 2b). In addition, integrin α6 (CD49f) was previously reported to be expressed in triple negative breast cancer cells [33] and, concordantly, positive staining for CD49f in the surface of MDA-1833 ​cells was observed (Fig. 2c). Therefore, surface marker expression was maintained inside our platform.

After morphological characterization of the bone tropic cells, the apoptotic profile of spheroids cultured inside the microfluidic was compared to spheroids grown in standard 96-well plates. No differences regarding spheroid size were observed (Fig. S2) and Annexin V staining showed that cell apoptosis was similar between conditions (Fig. 2d). We then investigated whether the environment in the microfluidic platform evoked changes in the conditioned medium of MDA-1833 ​cells. Breast cancer cells express a plethora of pro-inflammatory factors, of which interleukin (IL) 11 was previously implicated in breast cancer progression and bone metastasis [34]. IL-11 was therefore quantified and we observed a clear trend towards increased IL-11 levels inside the microfluidic compartment when compared to MDA-1833 cultured in standard 96-well plates (Fig. 2e). Thus, we further hypothesized that our microfluidic platform could recapitulate a more aggressive breast cancer phenotype. To confirm this, the conditioned medium from MDA-1833 spheroids cultured inside the microfluidic platform and in normal 96-well plates was collected and screened for the presence of proteins relevant for our model. The level of several matrix-associated proteins was increased in the conditioned medium from MDA-1833 spheroids cultured in our metastasis-on-a-chip platform, namely connective tissue growth factor (CTGF) and matrix metalloproteinase 1 (MMP1), which were already described to promote breast cancer progression in the bone niche (Fig. 2f) [31,[35], [36], [37]]. Additionally, multiple proteins previously reported to be involved in bone metabolism were shown to be more abundant when MDA-1833 spheroids were cultured inside the microfluidic platform when compared to 96-well plates, such as latent transforming growth factor-β (TGF-β) binding protein 1 (LTBP1) and Dickkopf-1 (DKK1) (Fig. 2f).

### 3.3 Sympathetic neuronal compartment: cell differentiation and catecholamine release

**Fig. 3:**
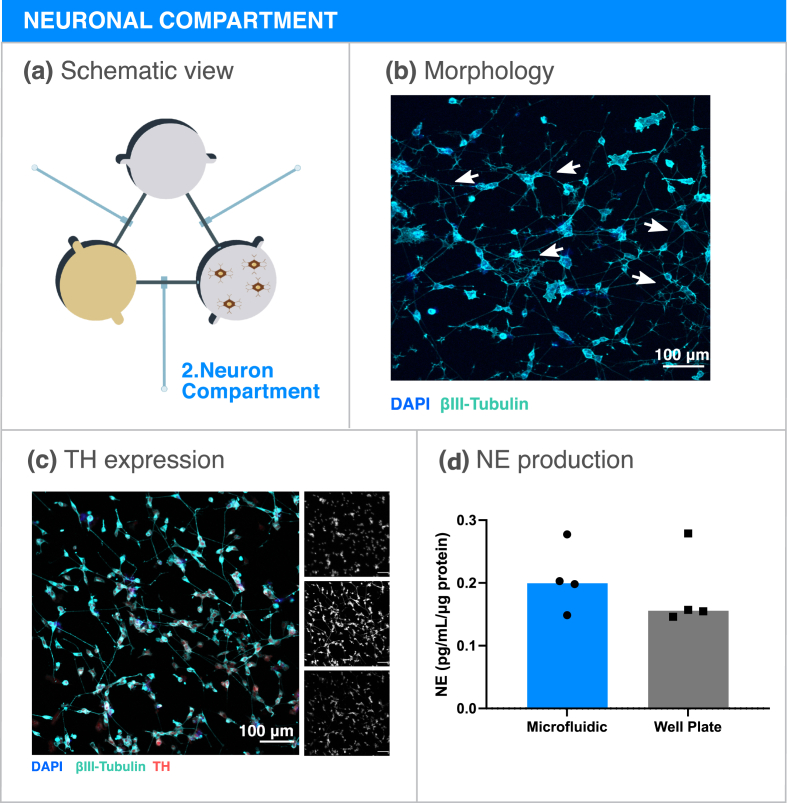
Neuron-like cell compartment optimization. (a) Schematic representation of the neuron-like cell compartment. (b) Micrograph of SH-SY5Y cells cultured for 7 days inside the microfluidic compartment. Several neuronal extensions are highlighted in white arrows. Blue - DAPI; Cyan - βIII Tubulin. Scale bar 100 ​μm. (c) Expression of the sympathetic marker TH in SH-SY5Y cultured in the microfluidic platform. Single channel images are showed on the right. Blue (Top) - DAPI; Cyan (Mid) - βIII Tubulin; Red (Bottom) - TH. Scale bar - 100 ​μm. (d) NE concentration quantification in SH-SY5Y conditioned medium from the microfluidic platform or in well plates. Data is expressed as median of individual data points from 4 independent experiments and was normalized to the total protein content (Mann-Whitney test, non-significant).

Sources for human sympathetic neurons for in vitro culture are scarce. Sympathetic neurons were previously obtained from human Pluripotent Stem Cells (hPSCs), however protocols for differentiation are inefficient and of difficult execution [38]. Nonetheless, neuroblastoma cell lines have been reported to produce NE [39], which is the main sympathetic neurotransmitter. In order to model the sympathetic nervous system contribution to bone metastasis, SH-SY5Y neuron-like cells were included in the microfluidic platform (Fig. 3a). The neuronal compartment was coated with laminin and SH-SY5Y cells were allowed to differentiate for 7 days under retinoic acid stimulation, after which they presented long axonal extensions (Fig. 3b). Tyrosine hydroxylase (TH) is the rate limiting enzyme in the NE synthesis cascade and commonly expressed in sympathetic neurons. TH expression was verified in SH-SY5Y cells cultured in the microfluidic platform, confirming that SH-SY5Y cells maintain a sympathetic phenotype when cultured in our metastasis-on-a-chip platform (Fig. 3c). Since NE secretion would be the main contributor to our metastatic model, NE was subsequently quantified in the conditioned medium of the neuronal compartment (Fig. 3d). NE was detected in a nanomolar concentration range, a concentration sufficient for adrenergic receptor stimulation [40]. No significant differences were observed between SH-SY5Y cells cultured in the microfluidic platform and the 96-well plate regarding NE production, validating the assumption that sympathetic input is maintained in our platform.

### 3.4 Bone compartment: bone resorbing osteoclasts on mineralized ECM

**Fig. 4:**
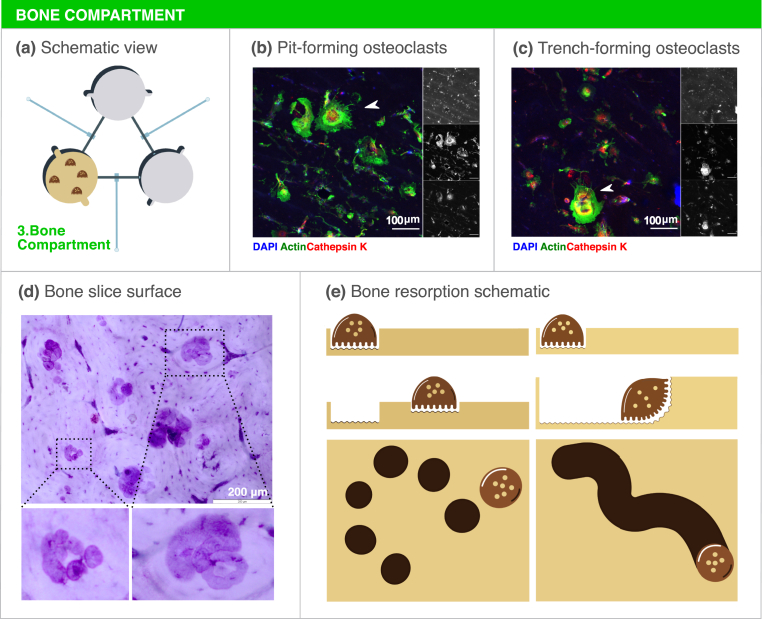
Bone compartment optimization. (a) Schematic representation of the bone compartment. (b) Micrograph of mature, multinucleated osteoclasts on the surface of the bone slice. The white arrowhead shows an osteoclast with a circular actin ring, characteristic of resorption pit formation. (c) Mature osteoclasts on top of a bone slice. The white arrowhead shows an osteoclast with a crescent shaped actin ring, characteristic of a resorption trench formation. Single channel images are showed on the right. Blue (Top) - DAPI; Green (Mid) - Actin; Red (Bottom) - Cathepsin K. Scale bar - 100 ​μm. (d) Toluidine blue staining of the surface of the bone slice after three days of culture. Several resorption events are seen throughout the bone slice. In the left inset resorption pits are visible while in the right inset an example of a trench is shown. Scale bar - 200 ​μm. (e) Schematic representation of osteoclast resorption activity. Osteoclasts are capable of stationary resorption (left) or resorption while moving through the bone surface (right), leading to the formation of resorption pits or trenches respectively.

Breast cancer is usually of osteolytic nature, leading to extensive bone degradation. Osteoclasts, multinucleated cells that are able to resorb the bone, are therefore crucial players in the establishment of metastatic bone lesions. In fact, proteins released from the bone matrix during resorption as well as other osteoclast-secreted factors are able to modulate breast cancer cell behavior and also neuron activation in the bone microenvironment [41,42]. Aiming to replicate these interactions, mature human osteoclasts were cultured on top of bone slices inside the microfluidic platform for three days, refreshing RANKL and *M*-CSF daily to ensure ample access to these cytokines (Fig. 4a). Characteristic features of mature osteoclasts were observed, namely large cytoplasm area, actin ring formation and osteoclast marker cathepsin K expression (Fig. 4b and c). Of note, different morphologies were observed in various osteoclasts, namely circular actin rings (Fig. 4b) or crescent-shaped actin rings (Fig. 4c), which reflect the direction of resorption and are characteristic of different resorption modalities [28,43]. Osteoclasts are inherently capable of resorbing the bone while being static or while moving across the surface of the bone, generating resorption pits or trenches, respectively (Fig. 4d, e, Fig. S3). Accordingly, resorption pits and trenches were visible on the surface of the bone slices, demonstrating that osteoclasts were not only morphologically differentiated but also fully functional (Fig. 4d).

### 3.5 Non-selective crosstalk: opening communication between breast cancer-neuron-osteoclast

**Fig. 5:**
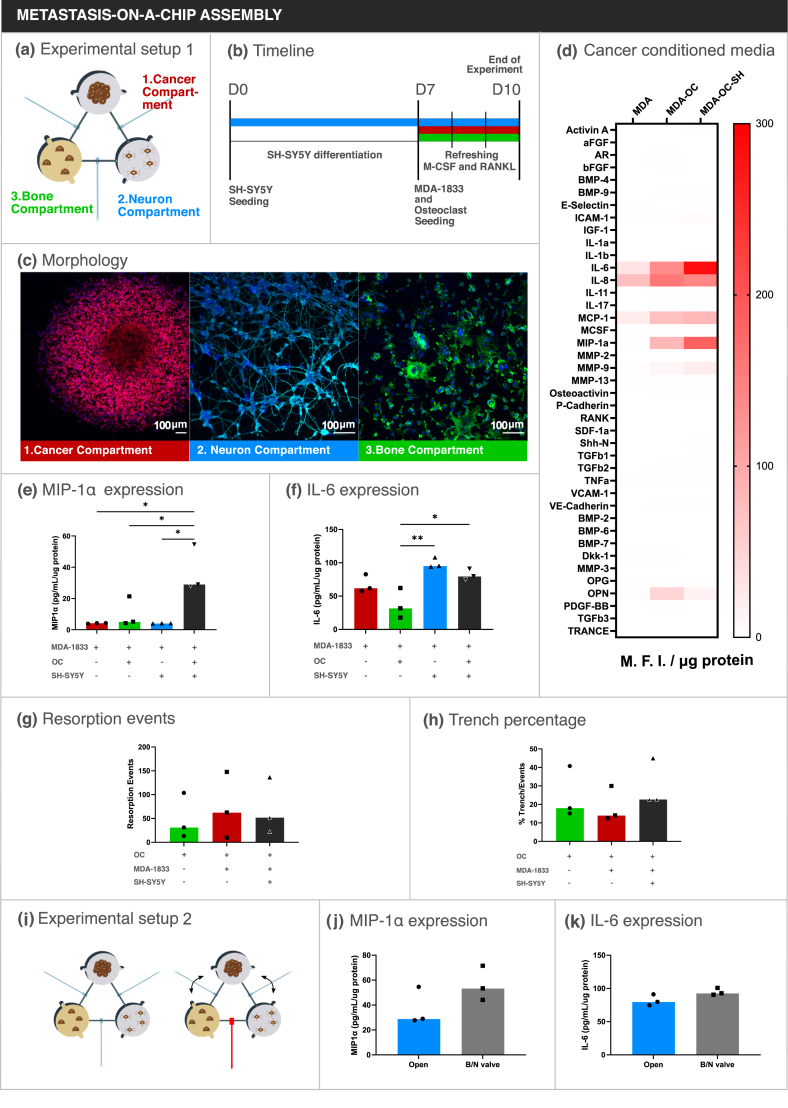
Tri-culture assembly on the microfluidic platform. (a) Schematic representation of the assembled microfluidic platform. (b) Timeline of the experiment. (c) Representative micrographs of (1) the cancer compartment, (2) the neuron compartment and (3) the bone compartment. Scale bar – 100 ​μm. (d) Bone metabolism array data of conditioned medium from the cancer compartment. Data is represented as Mean Fluorescence Intensity and normalized by total protein content. (e, f) Quantification of MIP1α and IL-6 concentration in conditioned media from the breast cancer compartment by ELISA. Data is expressed as median of individual data points from 3 independent experiments and was normalized to the total protein content (One-way ANOVA test, ∗p ​< ​0.05, ∗∗p ​< ​0.01). (g) Quantification of resorption event number and (h) percentage of trench number relative to total number of events. Data is expressed as median of individual data points from 3 independent experiments (One-way ANOVA test, non-significant). (i) Schematical representation of the experimental setting. Closing the valve between the neuronal and bone compartment forces communication to be preferentially through the cancer compartment. (j) Quantification of MIP1α and (k) IL-6 concentration in conditioned media from the breast cancer compartment by ELISA. Data is expressed as median of individual data points from 3 independent experiments and was normalized to the total protein content (Mann-Whitney test, non-significant).

After individual characterization of each compartment, MDA-1833 ​cells, SH-SY5Y cells and osteoclasts were cultured simultaneously in the microfluidic platform (Fig. 5a). To that end, SH-SY5Y were first seeded and differentiated with retinoic acid for 7 days, followed by osteoclast and MDA-1833 seeding in their respective compartments (Fig. 5b). Cells were further cultured for 3 days, refreshing RANKL and *M*-CSF daily in the bone compartment until the end of the experiment. Cell morphology of each cell type was confirmed by immunocytochemistry in the end of the experiment (Fig. 5c). This was an important validation step since we were able to show that similar morphology and differentiation status were achieved when all cells were cultured simultaneously, even though each compartment encompassed different culture media that were mixed by diffusion during the course of the experiment.

As stated above, the aim of this platform was to investigate how breast cancer cells would respond to sympathetic stimuli under osteoclast crosstalk, by focusing our study on breast cancer secreted factors. Since proteins described to modulate the bone microenvironment were detected in the conditioned media of MDA-1833 ​cells in the previous optimization step, we decided to restrict our search by using a bone metabolism protein array. Conditioned medium was extracted from the breast cancer compartment in the end of each experiment and screened for proteins that could be relevant for our model, e.g. IL-6 and monocyte chemoattractant protein 1 (MCP-1). After normalization for total protein content, the relative expression of each protein in the breast cancer compartment was determined (Fig. 5d). Of note, when breast cancer cells were co-cultured with osteoclasts, increased levels of IL-6, IL-8, MCP-1 and macrophage inflammatory protein 1α (MIP-1α) were observed in the breast cancer compartment, which point to an effect of osteoclasts on MDA-1833 secretome. Interestingly, the pro-inflammatory setting was further exacerbated when SH-SY5Y cells were added to the model, where the levels of IL-6 and MIP-1α were further augmented (Fig. 5d). Indeed, sympathetic stimulus has been described to increase IL-6 levels in MDA-231 breast cancer cells with high β-adrenergic receptor (β-AR) expression [44] and both MDA-1833 and human osteoclasts express several ARs, being therefore responsive to sympathetic stimuli (Fig. S4). Based on these results, MIP-1α and IL-6 were then selected as potential candidate targets of sympathetic signaling in breast cancer and proceeded to validate the array by quantifying the expression of these cytokines by ELISA. Again, we observed a significant increase in MIP-1α in the cancer compartment when all cell types were cultured together with the valves in an open state (Fig. 5e). Importantly, the levels of MIP-1α detected in the cancer compartment, when MDA-1833 ​cells were co-cultured with either osteoclasts or neurons, did not differ from MDA-1833 monoculture. These results highlight the potential of our microfluidic platform to capture the synergistic effect of multiple cell crosstalk and the importance of a dynamic interaction between the three cell types. On the other hand, IL-6 levels were significantly increased in the tri-culture condition when compared to MDA-1833 and osteoclast co-culture controls but were similar to MDA-1833 monoculture controls (Fig. 5f). The increase in IL-6 appears to be due to neuronal inputs, since the addition of SH-SY5Y cells sharply increased IL-6 production as could be seen in MDA-11833 and neuron co-culture controls. MDA-1833 ​cells are the main source of IL-6 since only residual IL-6 was detected in neuronal or osteoclast mono-culture controls (Fig. S5).

Despite the augmented levels of MIP-1α and IL-6 pro-inflammatory mediators in the cancer compartment, these did not affect overall osteoclast resorption activity nor resorption mode in the bone compartment (Fig. 5g and h).

Taken together, our results demonstrate the importance of the dynamic communication between the different players in the metastatic niche and the ability of our microfluidic platform to capture these complex interactions.

### 3.6 Selective crosstalk: closing communication between neurons and osteoclasts

Intercellular communication on the microfluidic platform is dynamic, where each different cell type is able to shape the response of the other cells over the course of the experiment. The inclusion of valves in our platform adds an extra layer of complexity, allowing the manipulation of the communication by stopping the flow between two specific compartments. The observed increase in pro-inflammatory factors in our model supported by other previous reports have shown that direct sympathetic stimulus increases breast cancer aggressiveness [44]. Thus, we hypothesized that the blockage of communication between the neuron and bone compartment would not affect the production of pro-inflammatory cytokines in our model. Taking advantage of the Quake valves incorporated in our metastasis-on-a-chip model, we assembled the tri-culture while keeping the valve between neuron and bone compartments closed, in order to assess the impact of different communication routes on the production of IL-6 and MIP-1α in the cancer compartment (Fig. 5i). As hypothesized, closing the valve between neuronal and bone compartment did not change the levels of MIP-1α nor IL-6 (Fig. 5 j, k). These results suggest that direct communication between SH-SY5Y cells and osteoclasts is not required for the observed levels of IL-6 and MIP-1α levels in the breast cancer compartment, since limiting communication between neuron and osteoclasts did not change the levels of these cytokines secreted by breast cancer cells.

## 4 Discussion

The crosstalk between the multiple components of the breast cancer bone metastatic niche is inherently complex. Although in vitro models provide a simplistic view of these intricate interactions, microfluidic systems can be used as versatile tools that are able to recapitulate important hallmarks of disease progression. Our work describes a new metastasis-on-a-chip platform designed to dissect the interplay between different cellular players within the breast cancer bone metastatic niche. This microfluidic platform retains a high degree of complexity by allowing the culture of at least three different human cell types simultaneously, namely bone tropic breast cancer cells, neurons and osteoclasts. Moreover, the physical separation of the cellular components enables a dynamic crosstalk between the different cell types exclusively through secreted factors, which will result in a cleaner readout interpretation, since direct cell-cell interactions do not occur in our model.

The majority of the microfluidic platforms used in the literature are produced using soft lithography processes that, although allowing a high degree of spatial resolution, also require costly facilities and highly qualified personnel. Using affordable consumer grade 3D printers, we were able to produce resin molds with adequate resolution and a smooth surface compatible with plasma cleaning bonding processes. Furthermore, the combination of features of different heights in the same mold facilitates rapid prototyping and can be translated into a design freedom that is not feasible in standard photolithography. For the first time, we describe a research tool that integrates human bone tropic breast cancer, neuron cells and osteoclasts in a single PDMS platform manufactured from 3D printed molds. We believe that this methodology could be employed in other research settings, since we used widely accessible 3D printers and computer aided design tools that do not require specialized training. Our platform was designed for the analysis of secreted factors involved in the crosstalk taking place at the bone metastatic niche, but compartment dimensions, channel length and cellular types could be prototyped and adapted to fit the needs of different biological questions.

Bone tropic MDA-1833 breast cancer cell spheroids were chosen to mimic a breast cancer bone metastatic niche. Cell spheroids are widely used to model tumor niche interactions and present advantages regarding metabolic gradients, apoptosis and drug resistance profiles when compared to standard monolayer culture techniques [45,46]. MDA-1833 ​cell spheroids were successfully introduced in the microfluidic platform and presented similar size and apoptosis levels as spheroids grown in normal 96 well plates. However, the environmental features inherent of the microfluidic compartment, such as lower access to nutrients and oxygen, seem to have an impact in MDA-1833 protein expression. Normalized IL-11 levels were shown to be increased in the metastasis-on-a-chip platform when compared to 96-well plates, coherent with a more pro-inflammatory and osteolytic phenotype. In addition, MDA-1833 ​cells were first described by Kang et al. and are reported to express a myriad of other osteolytic factors [31]. Of note, we showed that LTBP1 is more abundant in the conditioned medium of MDA-1833 ​cells cultured in the microfluidic platform when compared to normal 96-well plates. Osteoclasts are capable of cleaving LTBP1, which is subsequently involved in the release of TGF-β from the bone matrix during bone resorption [47] and will then fuel tumor growth in the bone niche. Furthermore, we showed that proteins from the matrissome [48], such as MMP1 and CTGF, were also increased in our microfluidic platform, consistent with an increased breast cancer aggressive behavior.

Since solid tumors are often of hypoxic nature due to limited or aberrant oxygen supply, hypoxia has profound implications in breast cancer progression and it has been already implicated in the establishment of bone metastasis [49]. Accordingly, the topographic features of our microfluidic platform imply a lower medium volume in the cell compartments and consequently a lower access to oxygen and nutrients environment when compared with a standard well plate. Hypoxia is described to promote the expression of CTGF in MDA-MB-231 ​cells [50], which might explain the observed increase in CTGF secretion.

Although we have focused on bone tropic breast cancer secreted peptides in our study, our microfluidic platform can be potentially used to analyze other secreted factors such as cancer-derived exosomes. Exosomes are extracellular vesicles that can deliver proteins, lipids and microRNAs to modulate cellular communication and have already been implicated in breast cancer bone metastasis and osteoclast differentiation [51]. In the future, this novel system can be potentially used to evaluate how sympathetic neuronal activation alters the secretion of exosomes by bone tropic breast cancer cells.

In order to study breast cancer associated bone pain, the crosstalk between sensorial neurons and breast cancer cells with microfluidic platforms has been previously modelled [52]. However, to our knowledge, no previous attempts have been made to model sympathetic neuron - breast cancer cellular interactions in a bone metastatic context using microfluidic technology. The culture of murine sympathetic ganglia in microfluidic platforms was established to study cardiomyocyte stimulation, but human sources of sympathetic neurons are limited. Retinoic acid differentiated SH-SY5Y cells have been previously used as models of sympathetic neurons [39], since these cells are capable of expressing the sympathetic marker TH and produce NE. In our model, we were able to successfully cultivate NE-secreting human sympathetic neurons, which was fundamental to achieve our final goal.

Previous microfluidic models, where murine osteoclasts derived from RAW264.7 ​cells were co-cultured with osteocytes to unravel the effect of mechanostimulation on the crosstalk between these cellular players, have been also already described [53]. However, as far as we are aware, our work is the first to describe the culture of human osteoclasts in a microfluidic platform. One of the advantages of our proposed model is the addition of bone slices to the bone compartment. Bone slices retain important topographic and biochemical cues that are crucial for osteoclastic resorption activity, allowing for resorption activity readouts that were not yet previously seen in microfluidic devices. Importantly, in addition to the total extent of osteoclast resorption activity, it is possible to distinguish different resorption modalities in the surface of the bone slices. Variations in the trench content relative to the total number of events could be indicative of differences in collagenolysis *versus* demineralization rates and cathepsin K activity [54]. In addition, trench resorption is faster and favors bone fragility, and is associated with more aggressive bone degradation [25].

After optimization steps, microfluidic assembly of all three cell types allowed a unique glance at the intercellular crosstalk that occurs at the bone metastatic niche. In our metastasis-on-a-chip platform, we showed that bone tropic breast cancer cells received synergistic inputs from neurons and osteoclasts that resulted in increased levels of pro-inflammatory cytokines. Interestingly, IL-6 and MIP-1α were already implicated in the progression of breast cancer bone metastasis and osteoclastogenesis [55,56]. Furthermore, our results are consistent with previous studies where sympathetic stimulus increases IL-6 production in breast cancer [44] and melanoma cell lines in vitro [57]. On the other hand, osteoclast secreted factors or proteins released from the bone matrix during resorption were also described to promote breast cancer growth [58,59]. As could be seen in MIP-1α secretion levels, we showed that dynamic communication, contrarily to standard conditioned medium approaches, is of vital importance to recapitulate interactions that could take place at the bone metastatic microenvironment.

One of the main features of our platform is the inclusion of valves in each of the interconnecting channels. These valves were designed based on the Quake valve architecture, where pressure applied to a flexible membrane allows the closure of a fluidic channel [60]. Quake valves were previously used in both photolithography [61] and 3D printing applications [16] and are a valuable tool for fluidic control. In this microfluidic platform, Quake valves serve two main purposes: 1) we were able to constrain each different cell in its respective compartment during cell seeding and 2) we could alter the diffusion pattern and directionality of the stimuli from one compartment to another. We demonstrated that by closing the communication between bone and neuron compartments, cytokine levels secreted by breast cancer cells did not change significantly, pointing towards a negligible effect of direct neuron-osteoclast communication on the changes observed in the cancer compartment. Although epinephrine was reported to increase differentiation of human osteoclast-like cells, the direct effect of NE on human osteoclast activity is still unknown [62]. Future effort should be directed towards understanding what are the osteoclast/neuron derived factors that promote a breast cancer pro-inflammatory phenotype.

Our model presents some limitations in its current form. First, we included sympathetic neurons and osteoclasts but the bone metastatic niche is extremely complex and composed of multiple cellular players [1]. We could potentially use our microfluidic platform to study how resident macrophages and lymphocytes, osteocytes, hematopoietic stem cells, fibroblasts and endothelial cells could also contribute to the establishment and progress of the metastatic disease. For instance, future improvements to this microfluidic model would include the addition of immune cells to the cancer compartment. Since macrophages are responsive to NE [63], it would be interesting to assess how they impact breast cancer bone metastasis under sympathetic stimulus. In addition, under sympathetic stimulation, osteoblast derived vascular endothelial growth factor (VEGF) and IL-1β was able to modulate endothelial cells to facilitate breast cancer cell extravasation from circulation into the bone marrow niche, both in vitro and in vivo [18,22]. A different platform design would be required to include endothelial cells, though, to allow hydrogel seeding, fluidic flow and self-assembly of blood vessels.

Second, several studies have shown that breast cancer cells induce osteolysis via osteoblast-lineage cells and not by direct osteoclast stimulation [64,65]. Parathyroid hormone-related protein (PTHrP) is described to be secreted by breast cancer cells and to promote the production of RANKL by osteoblasts, subsequently resulting in osteoclast activation [65]. Furthermore, the secretome from MDA-MB-231 ​cells was recently described to modulate osteoblast mediated bone matrix deposition in vivo [66]. On the other hand, osteoblasts were observed in close contact with prostate and breast cancer cells in clinical bone metastasis biopsies, suggesting that not only secreted factors but also direct cell-cell contact between osteoblasts and cancer cells could be important for osteoclastogenesis and bone degradation [67]. The addition of primary human osteoblasts in the cancer compartment, mimicking the interactions that take place between tumor cells and cancer associated fibroblasts, could be an interesting upgrade to the microfluidic model in order to fully recapitulate the bone resorption promoting capacity of breast cancer cells.

Third, the potential of our platform to investigate the effect of different cancer cell lines on the bone niche could be further explored. We focused on metastatic MDA-1833 breast cancer cells to mimic the local colonization of bone, nevertheless it would be interesting to assess how the parental cell line MDA-MB-231 react to osteoclast secreted factors under sympathetic activation. Furthermore, differences in the role of distinct molecular subtypes of breast cancer on the bone niche, such as luminal-like or epidermal growth factor receptor 2 (HER2) enriched breast cancer cell lines, could be investigated in future studies. Finally, prostate cancer, lung cancer and multiple myeloma also display bone tropism [59], and thus studying the effect of sympathetic stimuli on prostate/lung or multiple myeloma bone metastatic niche would demonstrate the translational potential of our model.

## 5 Conclusion

In summary, we have developed a new model of sympathetic regulation of breast cancer in a bone metastatic context through the integration of breast cancer cells, osteoclasts and sympathetic neuron-like SH-SY5Y cells on the same microfluidic platform. Our model displays several advantages compared to other breast cancer metastatic models: 1) manufacture is cheap and accessible without the need for specialized staff and equipment; 2) integrates a full humanized system with bone tropic breast cancer cells; 3) contains a bone matrix with intact biomechanical and biochemical cues leading to relevant bone resorption readouts; 4) includes three quake valves that allow compartment isolation during cell seeding and modulation of diffusion directionality. We successfully characterized the culture of different cell types on our platform, which led to the identification of inflammatory mediators that could be involved in the breast cancer response to sympathetic stimuli in the context of bone metastasis.

Collectively, our findings could set the basis for additional exploration of the mechanisms that govern sympathetic modulation of breast cancer bone metastasis. Further insights on the cancer-bone-neuron crosstalk could be gained by manipulating the microenvironment of each compartment by the addition of different cell players, the use of specific signaling pathway inhibitors or by including breast cancer knock-out variants. In the future, our metastasis-on-a-chip platform could also potentially be applied for drug screening assays and incorporate patient-derived tumor samples or osteoclasts for improved translational relevance. Importantly, this work presented a new platform that could be used as basis for fundamental research on various physiological and pathological settings, with the possibility of changing the different cell types to cater the needs of individual research questions.

## Credit author statement

**Francisco Conceição**: Conceptualization, Methodology, Validation, Formal Analysis, Investigation, Writing - Original Draft, Visualization. **Daniela M. Sousa**: Investigation, Writing – Review & Editing. **Joshua Loessberg-Zahl**: Methodology, Writing – Review & Editing. **Anke R. Vollertsen**: Methodology, Writing – Review & Editing. **Estrela Neto**: Investigation, Writing - Review & Editing. Kent Søe: Supervision, Writing – Review & Editing. **Joana Paredes**: Supervision, Writing – Review & Editing. **Anne Leferink**: Resources, Supervision, Conceptualization, Writing – Review & Editing. **Meriem Lamghari** – Supervision, Project Administration, Funding Acquisition, Writing – Review & Editing.

## Data availability

The data that support the findings of this study is available from the corresponding authors upon reasonable request.

## Declaration of competing interest

The authors declare that they have no known competing financial interests or personal relationships that could have appeared to influence the work reported in this paper.
